# Catalytic Activity and Kinetic Modeling of Various Modules HZMS-5 and Treated MCM-41 Catalysts, for the Liquid-Phase Ketalization of Glycerol With Acetone

**DOI:** 10.3389/fchem.2019.00799

**Published:** 2019-11-26

**Authors:** Murad Alsawalha

**Affiliations:** Department of Chemical and Process Engineering Technology, Jubail Industrial College, Al Jubail, Saudi Arabia

**Keywords:** kinetic study, ketalization reaction, reaction rate, HZSM-5 zeolites, MCM-41, glycerol

## Abstract

Studies of the Ketalization reaction using trivalent alcohol glycerol in combination with acetone and their kinetics modeling are still limited. The focus of this current study is an investigation into HZSM-5 with various silica to alumina molar ratios (M = 35, 90, and 160) for the reaction between glycerol and acetone. In addition, the influence of reaction temperatures (25, 50, and 60°C) on the rate of the reaction have also been considered. Additionally, this investigation established the rate law for all HZMS-5 models (M = 35, 90, and 160) which showed “*n*” order equals half while the activation energy was found to be 164.34 kJ mol^−1^ with a constant reaction rate of k_0_ = 5.2678^*^10^28^ (Concentration^1/2^. min-^1^). Furthermore, MCM-41 pure mesoporous materials were separately treated using various methods. The first involved treatment using Dichlorodimethylsilane MCM 41(TD) and later treatment of a pure sample with sulfuric acid MCM-41. The sulfated MCM-41 sample (MCM41-SU) showed that reaction order equals *n* = −1 and a rate constant of (k) = 3.9 × 10^2^ (Concentration^−2^. min^−1^). A close correlation and agreement was found between the experimental modeling and the theory. Additionally, this current kinetic study showed that water production has no effect on the conversion activity within 10 min from the start of reaction. Besides, further kinetics investigations were performed to ascertain the estimated time for water production based on the conditions applied during the reaction system. It resulted in an average time of 3 min for equilibrium to be reached in the reaction system. It was found that the estimated reaction equilibrium time (t_*eq*_) is within the range from zero to 10 min in agreement with the proposed kinetic model in this work. Finally, it was also observed that a low equilibrium conversion (X_Aeq_) had been obtained in the present work about 0.42 (42%). At a reaction temperature of 60°C (333.15 K) and at one atmosphere, the acetone was shown to exert a vapor pressure of about 113.737 mm Hg. Hence, the overall order of the reaction was determined by the method of initial rates. Similarly, in order to ascertain the dispersion of aluminum, together with its distribution on the surface of a catalyst for a zeolite that has varying molar ratios of silica to alumina as is the case for example with ZSM-5 (35), a mathematical approach is proposed in this study for its calculation.

## Introduction

Zeolite materials have an important role in terms of their applications in the various industries (Alsawalha, 2019; Pan et al., 2019). There are a variety of definitions in use which do not differ significantly, but the most commonly accepted is by Breck (1974). Zeolites are crystalline, hydrated aluminosilicates, synthesized or naturally occurring which contain alkali or alkaline earth cations, namely, sodium, potassium, magnesium, calcium, strontium, and barium.

**Figure 1:**
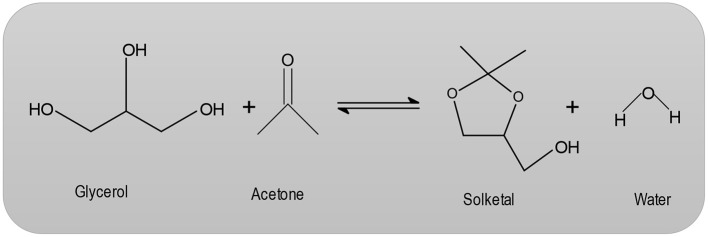
Condensation reaction between glycerol and acetone.

Structurally, zeolites are Aluminosilicate frameworks based on an infinite, three-dimensional network of AlO_4_ and SiO_4_ tetrahedrals interconnected by sharing oxygen atoms (Corma et al., 1997; Loganathan et al., 2013). The synthesis of MCM-41 occurs via organic amphiphiles (e.g., quaternary ammonium salts), which are used as structure-directing reagents (Loganathan et al., 2013). Beck et al. (1992) describe the production of MCM-41 using the LCT mechanism (liquid crystal template). These MCM-41 materials are forms of liquid-crystalline phases of water/ surfactant systems and the periodic pore systems of the M41S group (Beck et al., 1992). The MCM-41 with periodic cavities can be synthesized with pore diameters ranging from 1.5 nm to over 10 nm (Beck et al., 1992). Heterogeneously catalyzed dehydration reactions have long been known. However, the mechanism of these types of reactions are not fully understood. Pines and Pillai (1960) suggest that both acidic and basic centers are required for catalyzed dehydration reactions. With regard to zeolites, the dehydration of alcohols on an HZSM-5 has been studied in particular (Derouane et al., 1978; Jingfa et al., 1988; Schulz and Bandermann, 1994). The literature describes the application of H-zeolites for the condensation reaction (Clarkson et al., 2001; Figure 1). In the heterogeneously catalyzed condensation reaction between glycerol and acetone, the catalytic condensation of glycerol with acetone leads to the formation of 2, 2-dimethyl-4-methanol-1,3-dioxolane, which is commonly referred to as solketal (Clarkson et al., 2001).

Solketal is a ring-shaped diether that has an additional hydroxyl group. The first developments of this synthesis began in the 1920s, when Fischer et al. (Fischer and Pfähler, 1920) devoted themselves to the study of a variety of glycerides. Further studies employing hydrochloric and sulfuric acid were performed (Fischer and Pfähler, 1920), but the preferred catalyst in the field of research is p-toluenesulfonic acid (Newman and Renoll, 1945). There is a dramatic and ever increasing interest in glycerol production from biodiesel, since it is manufactured as a by-product of fatty acid methyl ester production. The need for crude glycerol does not increase in proportion to the increase in the volume of biodiesel in production, resulting in its loss of value. Moreover, the literature (Rossa et al., 2017) presents a possibility of further processing glycerol which may then be added to various fuels as oxygenate. Thus, the trihydric alcohol could be converted either to acetone or to a compound, which also has a carbonyl group. Ketalization product, Solketal could be then added as an additive in gasoline, diesel, or biodiesel to increase ignitability and reduce particle emissions.

The challenge that water, a coproduct, appears in the reaction is based on the fact that glycerol and acetone form a heterotopic mixture and there is a need for the removal of the water from the equilibrium reaction (Fischer and Pfähler, 1920).

**Figure 2:**
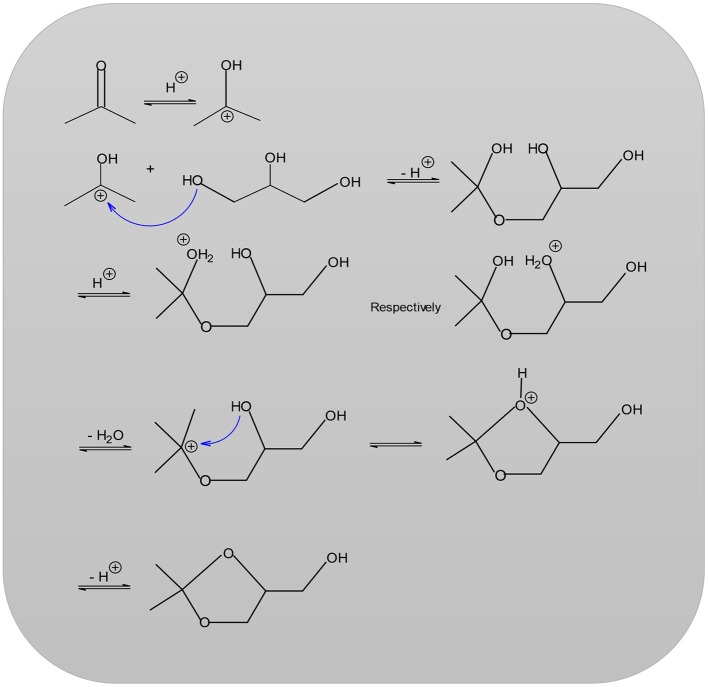
Mechanism for the formation of solketal reaction.

When feasible, it is possible to use a desiccant in the removal of water such as sodium sulfate, potassium carbonate or phosphorus pentoxide. However, employing desiccant has the disadvantage that on a production scale, waste products are obtained in large quantities, especially water (Fischer and Pfähler, 1920). The reaction temperature can range anywhere from room temperature up to the boiling point of acetone (56°C). Under the influence of acid catalysts, ketones react with polyhydric alcohols to form ketals (Nanda et al., 2016; Zong et al., 2018; Figure 2). In the first step of the reaction (Nanda et al., 2016; Zong et al., 2018), the acetone carbonyl group is activated by a coordination of the Lewis acid metal sites. Then, an alcoholic (hydroxyl) group of glycerol attacks a carbon atom within the carbonyl group. This attack coincides with the bond formation of an atom of carbonyl oxygen with a secondary one of carbon glycerol. Lastly, the dehydration process results in the product Solketal.

In the first step of the reaction, the acetone carbonyl group of the acetone is protonated and the secondary carbenium ion obtained is later attacked (nucleophilic) by a hydroxyl group of the glycerol before the final elimination of a proton. The newly formed hydroxyl group of the acetone or the mediate hydroxyl group of glycerol is affected because of protonation and the fact that water splits off to form another secondary carbenium ion. An intermolecular nucleophilic attack by a hydroxyl group leads to the desired product solketal (García et al., 2008; Nanda et al., 2016). The solketal reaction is selected for this work so as to examine the catalytic activity as well for performing a kinetics study over H-ZSM-5 and treated MCM-41 samples. There are few studies or references available in the literature regarding the study of kinetics using solketal reaction in particular for the MCM-41 samples. For this reason, the entire focus of this work is to establish a kinetics study for the ketalization reactions of both HZSM-5 (with various modules 35, 90, 160) and MCM-41. These solid-state acids are investigated with respect to a possible relationship between the modulus and the catalytic activity. Moreover, a sample of the pure, original source of MCM 41 was treated with Dichlorodimethylsilane and another with Sulfuric acid. After treatment, the first is named MCM 41 (TD) while, the second sulfated sample is named MCM 41 (SU) and both are included in this current work.

Samples were examined for both catalytic activity and for establishing reaction kinetics modeling. As is known to date, kinetics modeling in particular for HZSM-5 and MCM-41, have not yet been investigated by the Ketalization reaction. Accordingly, the current investigation seeks to contribute in the exploration of knowledge of mathematical and kinetics materials chemistry and its development and potential use in future industrial applications.

## Materials and Methods

### MCM-41 Silylated With Dichlorodimethylsilane (MCM 41-TD)

Purely siliceous MCM-41 (pure MCM-41), glycerol and acetone (99.5%) were purchased from Merck-Aldrich. Further modification of the (MCM-41 pure) was conducted in two stages. Twenty grams of (MCM-41 pure) was first silylated with 20 (mL) Dichlorodimethylsilane (99.5%) to produce a hydrophobic surface and stirred continuously at room temperature in a fume hood for a period of 1 h. Finally, the mixture was filtered and was left exposed in the open air for a period of 24 h again in a fume hood. At a later time, the preparation was heated in a furnace at 120°C for a period of 2 h. For each Ketalization reaction, an amount of 2 grams of catalyst material was used.

### Sulphated MCM-41 (MCM 41-SU)

A 15 gram sample of commercial MCM 41 samples purchased from Süd-Chemie AG, München was calcined for 2 h at 150°C, after which 20 milliliters of dichloromethane was added. This solution was then mixed magnetically whilst gradually adding drops of sulfuric acid measuring 10 milliliters were gradually added over a period of 2 h. The preparation continued to be magnetically stirred until the H_2_SO_4_ had been removed. Next, the sample was washed in 25 milliliters of acetonitrile and left to dry at room temperature to obtain Sulphated (MCM 41-SU). For the Ketalization reaction, an amount of 2 grams of catalyst was utilized.

### Catalyst Activation

A list of companies and zeolite types that were sourced for the study are presented in Table 1.

**Table 1:** Different commercial samples.

**Zeolite type**	**Manufacture**
ZSM-5 (Si/Al = 35) and ZSM-5 (Si/Al = 160)	The PQ corporation Valley Forge, Philadelphia
ZSM-5 (Si/Al = 90)	Süd-Chemie AG, München
p-Toluenesulfonic acid	Merck

It is noteworthy to mention that ZSM-5 zeolites number in the parenthesis [(SiO_2_)x(Al_2_O_3_)y] corresponds either to Si/Al ratio or the ratio of the oxides, so called module, SiO_2_/Al_2_O_3_ (this value is twice as high as the Si/Al ratio).

Zeolites have the capacity to absorb increasingly larger amounts of water from the air when stored for extended periods of time. Such a situation causes blockages in both their pores, as well as the active centers. Therefore, in order to enhance their activity, zeolites must be thermally treated before use (Corma et al., 1998; Konno et al., 2014; Jelena et al., 2015; Joris and Bert, 2018). In this process of treatment, any remaining ammonium compounds that were used as templates in the synthesis are removed.

With the decomposition of these zeolite molecules, ammonia and protons are formed (Corma et al., 1998; Jelena et al., 2015).

Before any zeolite could be used, it was important to have activated them first by using a tube furnace, for example. Thus, glass spoons were employed to introduce the catalyst samples into the glass tube. Then, in an atmosphere (I bar) of nitrogen, the oven was heated at 18°C per min to the respective final temperature of 350°C. Gauging correct temperatures and time duration is necessary for successful activation. These however, also depend on the catalyst system used. For example, the Zeolite HZSM-5 needed to be heated at 400°C for 1 h. Whilst, the MCM-41, whether in pure or modified form, required a temperature of only 130°C, but for an extended period of 8 h. Note that the reaction temperatures for all MCM-41 samples were at room temperature.

However, for determining the temperature influence on the kinetics behavior using HZSM-5 (M = 90) to assess how zeolite successfully converts a reactant to a Solketal product, two additional reaction temperatures of 50 and 60°C were applied.

### Condensation Reaction of Glycerin and Acetone

The homogeneous or heterogeneously catalyzed reaction between glycerol and acetone was carried out in a batch process. The reaction vessel used was a 50 mL, three-necked flask equipped with a thermometer, a reflux condenser, and a drying tube together with 8 grams of glycerol, 20 mL (20 × 0.784 = 15.68 g) of acetone and 0.3 mL of 1,4-dioxane. The implemented conditions for the kinetics investigations were determined at 600 rpm, a molar ratio of glycerol: Acetone, is $\left(\frac{8}{92.1}:\frac{15.68}{58.1}=0.087:0.27=1:3\right)$, and 4 w% (0.32 g) of catalyst relative to the mass of glycerol, for 100 min.

The 1,4-dioxane served as an internal standard for gas chromatographic analysis. In addition, an internal standard was also used because it was not possible to detect glycerol. As an example, acetonitrile can be used as a solvent on the analysis samples because it has the quantitative ability to dissolve both glycerol and acetone.

However, the disadvantage is that retention times of the reactant acetone do not differ from those of acetonitrile. This then prompted the use of an internal standard. However, there are usually no resulting negative effects of the use of acetone nor any influence on the process or results of the study. Additionally, the use of any alcohol was excluded as a solvent because it reacts adversely to the acetone.

### GC Conditions of Analysis

The various analyses were performed with the help of gas chromatography (GC) analysis type Perkin Elmer Auto system with flame ionization detector (FID). The temperature of the GC detector was 200°C under a gas stream of 35 mL/min H_2_. The applied carrier gas of H_2_ reached a pressure of 60 kPa. The GC column type is Carbowax (30 m × 0.25 mm × 0.25 μM polyethylene glycol). Temperature program of the gas chromatography began at 75°C isotherm for 1 min. It was raised gradually by 40°C per min up to 150°C. Thereafter, the same temperature was maintained for a further 2 min. The retention time of the detected compounds, acetone, 1,4-Dioxane, and the solketal product were found to be 1.2, 1.4, and 1.9, respectively. No other additional compounds have been detected.

An internal standard was employed to determine the condensation reaction. The peak area is multiplied with the correction factor of the internal standard. This represents 100% of the reactant peak areas largely because acetone, not glycerol, was identified. The correction factor that results can be calculated in the following way;

$$\begin{array}{l} Cr=\frac{P_{Ac}}{P_{In\;}.M_{ex_{ac}}\;} \end{array}$$

Cr, Correction factor

*P*_*Ac*_, Peak area of the acetone in the chromatogram

*P*_*In*_, Peak area of the internal standard in the chromatogram

*M*_*e*_*x*__*ac*__, Molar excess of acetone in the reaction mixture.

The correction factor for the conversion calculation of the condensation reaction between glycerol and acetone was calculated using the internal standard 1,4-dioxane. This was determined by means of the peak areas detected by an FID. However, the molar excess of acetone had to be considered.

The following is an example calculation for determining the correction factor. The detected peak are of acetone gave a value of 108, 680,851 and the 1,4 dioxane (internal standard) of 260,805 resulting in the following factor.

Example:

$$\begin{array}{l} Cr=\frac{P_{Ac}}{P_{In\;}.M_{ex_{ac}}}\\ Cr=\frac{108680851}{260805\;*\;3\;}\\ Cr=\;138.90 \end{array}$$

then,

$$\begin{array}{l} Cr.P_{In\;}=A_{100\%} \end{array}$$

where; *A*_100%_ is the 100% of the peak areas resulted from the chromatogram.

As a result, the conversion can be calculated from the following equation;

$$\begin{array}{l} X=1-\;\frac{P_{Ac}}{A_{100\%}}=\frac{A_{s}}{A_{100\%}} \end{array}$$

where *A*_*S*_ is the peak area of the Solketal.

### Surface Area and Pore Volume Characterizations

In the study, Tristar 3000 Surface Area and Porosimetry Analyzer (Micromeritics) equipment was employed to analyze different HZM5 and pure MCM 41 zeolites. Calculations of specific areas, volumes, and pore sizes were performed. Firstly, the BET (Brunauer, Emmet, and Teller) method determined the specific area. Secondly, the BJH method was used to ascertain the specific volume together with pore diameters. Third, the samples were weighed, then dry heated for 14 h at 350°C in a 6 × 10^−3^ vacuum. Following cooling at room temperature, the samples were then weighed a second time and later exposed to a temperature of −196°C. The sorption (adsorption/desorption) isotherms of N_2_ at different partial pressures of N_2_ were then ascertained. Microporous structures, consistent with zeolite materials, were noted.

## Results

Table 2 presents the results for different zeolite samples. Results revealed that zeolite H-ZSM5 possesses a higher specific area. This is likely due to a greater occurrence of micropores and external area specifically pore volumes together with mesopore volume of 0.07–0.13 [cm^3^ g^−1^]. In general, these findings are as expected for microparticles HZSM-5 (Nda-Umar et al., 2018).

**Table 2:** Textural properties of various HZSM-5 with different of SiO_2_/Al _2_O_3_ ratios.

**HZSM-5 with different SiO_**2**_/Al _**2**_O_**3**_ ratios**	**Micropore volume(cm^**3**^g^**−1**^)**	**Mesopore volume(cm^**3**^g^**−1**^)**	**Total pore volume (cm^**3**^g^**−1**^)**	**BET surface area SBET(m^**2**^g^**−1**^)**
ZSM-5 (35)	0.05	0.02	0.07	388.4
ZSM-5 (90)	0.06	0.04	0.1	425.6
ZSM-5 (160)	0.08	0.05	0.13	460.3

Moreover, the content of Na_2_O (%) in all commercial ZSM- 5 samples is <0.2. All ZSM-5 commercial samples have been calcined in an oven at 450°C for 5 h with a ramp of 15°C/min. The samples were later stored in an oven at 120°C in order to avoid contact with water. Conversely, Table 3 presents the textural properties of various MCM 41 samples.

**Table 3:** Textural properties of various MCM-41 samples.

**MCM-41 samples**	**BET surface area SBET(m^**2**^g^**−1**^)**	**Total pore volume(cm^**3**^g^**−1**^)**	**Average pore diameter (nm)**
MCM 41 (pure)	820	0.69	2.50
MCM 41 (TD)	988	0.76	2.54
MCM 41 (SU)	1,382	1.10	2.81

In this study, findings concerning the characterization of the surface area of the pure commercial siliceous MCM-41 (pure MCM-41) were in the range of 820 (m^2^g^−1^), with pore volumes of 0.76 (cm^3^g^−1^) and pore diameter measuring 2.5 nm. On the other hand, higher surface area was recorded for sulfated sample (MCM 41-SU) in the range of 1,382 (m^2^g^−1^), with total pore volume of 1.1 (cm^3^g^−1^) and pore diameter with the value of 2.81 nm. It is possible that treatment with sulfuric acid precipitates changes in the MCM 41-SU. The treatment process itself may have resulted in an unexpected higher pore volume of MCM 41-SU, possibly due to the partial deterioration of walls of the mesopores.

Additionally, this higher surface area of the MCM-SU is likely to be a result of the acidic species of the sulfuric acid that were grafted onto the surface of Si-MCM-41 in the treated sample.

Likewise, the literature details how purely siliceous MCM-41 was treated with Phosphorus acid for a shortened period (Kawi et al., 2002). The results showed that the surface phosphorus species had grafted onto the surface of MCM-41 and that an increase in the number of selectively formed Brønsted acid sites on the surface had also occurred without the formation of Lewis acid sites (Kawi et al., 2002).

In the future and as a follow on from our study, more investigation is required in order to examine in greater detail the effects of various acidic treatments over the surface areas for the samples studied.

### Reaction of Glycerol and Acetone Using p-toluenesulfonic Acid as a Homogeneous Catalyst

**Figure 3:**
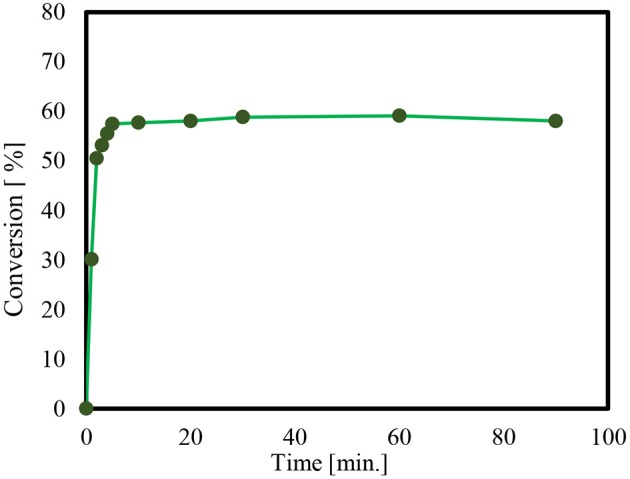
Reaction of glycerol and acetone using p-toluenesulfonic acid (PTSA), as a homogeneous catalyst, at 60°C.

In order to check if Solketal takes form in the absence of a catalyst, an additional “unanalyzed” reaction was performed. It was noted that no product could be detected using Gas chromatography during the 24-h period. Thus, the time it takes for product formation is only attributable to the activity of the catalyst employed. Moreover, the reaction of glycerol and acetone by p*-*toluenesulfonic acid (PTSA), as a homogeneous catalyst is presented in Figure 3.

The conversion of the condensation reaction between glycerol and acetone on different p-toluene sulfonic acids (PTSA) (Breck, 1974; Alsawalha, 2019; Pan et al., 2019) as a homogeneous catalyst showed about a 38% catalytic activity 10 min after the start of the reaction. The maximum conversion of 58% was reached within 30 min. The conversion results obtained in this present work is in agreement with the work in literature (Nda-Umar et al., 2018), where a molar ratio of 1:4, conversion is 70.9%, and at a molar of 1:2, the conversion is 54.9% using similar catalyst. That is to say, the conversion of glycerol at 1:3 molar ratio of glycerol to acetone in the present study is within the range 58%.

The obtained result is also an agreement with the documented conversion of glycerol to acetone over p-toluene sulfonic acid (PTSA) which reached 60% after 15 min, increasing to 80% after 45 min and with a reaction temperature 70°C (Da Silva et al., 2009). For an objective comparison of this current result with that found in the literature, note that the different conversions of glycerol were found to reach to reach 87 and 79%, over acid catalysts like H-β zeolite and Zr(SO_4_)_2_, respectively, and at a reaction temperature of 40°C (Nanda et al., 2014a). Notably, water as by-product forms and appears to impede the acetalization reaction (Smirnov et al., 2018). It acts as a barrier to the successful conversion of glycerol, a kind of thermodynamic and kinetic obstacle. It is likely that the presence of water, even in very small amounts resulted in only a modest conversion of 58%. It also increased the need for longer reaction time so as to manage and lessen the effect of water forming in the pores (Smirnov et al., 2018).

On the other hand, for acetalization of 2.74 mol glycerol with 8.22 mol acetaldehyde, one study employed 0.27 mol p-toluenesulfonic acid as a catalyst (García et al., 2008). The reaction time was for 16 h as the reflux was heated. This resulted in a 90% yield of the product 2,2-dimethyl-1,3-dioxolan-4-yl methanol (García et al., 2008).

### Heterogeneous Catalyzed Condensation

#### Zeolites

**Figure 4:**
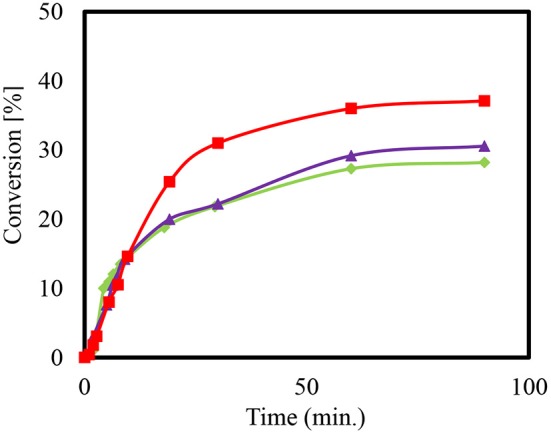
Conversion (%) to Solketal using HZSM-5 with different ratio (SiO_2_/Al_2_O_3_); 35 (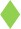), 90 (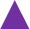), and 160 (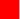), at reaction temperature 50°C.

The conversion of the condensation reaction between glycerol and acetone on different zeolites was investigated. The acidity of zeolites is dependent on the ratio of silica to alumina. The smaller the value of the module, the more that Brønsted acid sites are present. Moreover, the medium-pore zeolite of ZSM-5 was added to the condensation reaction in the H form with the modulus values 35, 90, and 160. Figure 4 shows the presented conversions over time.

When using the heterogeneous catalyst HZSM-5 (M = 90) it can be observed that more time is needed to reach the maximum conversion of about 38% compared to HZSM-5 with modulus (SiO_2_/Al_2_O_3_) value (M = 35). The final set of conversions that were realized are estimated to be as equivalent since they are within the range of the measuring accuracy of the sampling and that of the gas chromatographic analysis. This equivalency may be explained by changes in the acid center density without affecting the conversion of the reaction (Rossa et al., 2017). Moreover, lower conversions for the condensation reactions using HZSM-5 compared to the homogeneously catalyzed experiment may be attributed to the reaction anhydrous system (Da Silva and Mota, 2011). The preexisting water molecules that formed can block the catalytically active centers of the zeolites, which would result in a lower efficiency. The literature reports a higher conversion with zeolite beta in the range of 90 and 95% (Da Silva and Mota, 2011). Moreover, the selectivity of the Solketal product obtained for all tested samples yielded around 98%, which is in agreement with results in the literature (Rossa et al., 2017).

#### Temperature Influence

**Figure 5:**
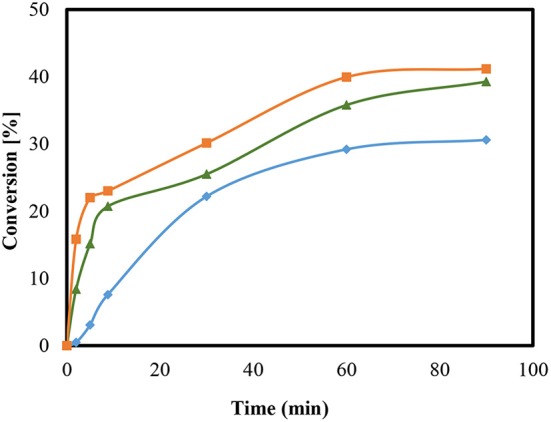
Conversion of the condensation reaction using HZSM-5 with a modulus of M = 90 at temperature of 25°C (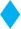), 50°C (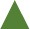), and 60°C (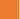).

To investigate a possible influence of the temperature on the reaction, an examination was carried out on HZSM-5 (M = 90) at 25, 50, and 60°C as presented in Figure 5. The amount of catalyst used was 5 wt.%.

Variations in temperature were found to possibly influence the conversion. After 90 min, the conversion at 25°C was 20.7% and at a temperature of 50°C, the conversion raised to 39% at 60°C, a slight increase in conversion was observed about 41%. The study results obtained here are also in agreement with the figures presented in the literature (Rossa et al., 2017). The alteration in the reaction temperature using H-BEA zeolite and the same reaction of glycerol with acetone stimulated a positive impact on conversions (Rossa et al., 2017). In addition, other results from Kowalska-Kus et al. (2016) showed that the conversion over HZSM-5 increases as the particle size of the catalysts decrease (Kowalska-Kus et al., 2016). Moreover, no further investigations were possible at higher temperatures because of the low boiling point of acetone. Additionally, the use of open equipment and the extension of the sampling time would likely lead to a reduction in the content amount of acetone and so cause distortions in any further comparative analysis.

Furthermore, an earlier study of the application of zeolites was conducted by Da Silva et al. (2009). In this, conversion of glycerol to fuel-additives was investigated with the use of zeolite Beta and p-toluene sulfonic acid and with the addition of acetone and formaldehyde. Moreover, mesoporous Lewis acid catalysts could be active in acetalization of glycerol with acetone to produce solketal (Da Silva et al., 2009). A five-membered-ring solketal was proposed in the acetalization of acetone with glycerol and for catalyzation with solid Brønsted acids (Li et al., 2012). By proper coordination and activation of the carbonyl group of the acetone, the study (Li et al., 2012) contended that Lewis acid metal sites could function in similar ways as those oxidation reactions shown in Meerwein–Ponndrof–Verley reduction studies (Li et al., 2012). In the same instance that the primary alcoholic group of glycerol attacks the carbon atom of the carbonyl, a bond is formed between the carbonyl β-carbon and oxygen atoms. The resulting dehydration precipitates the formation of a five-membered-ring solketal (Li et al., 2012). Moreover, literature (Rossa et al., 2017) reported that during the reaction, it is almost certain that water was formed. As a result, the structure of the H-BEA zeolite destabilized (Li et al., 2012).

**Figure 6:**
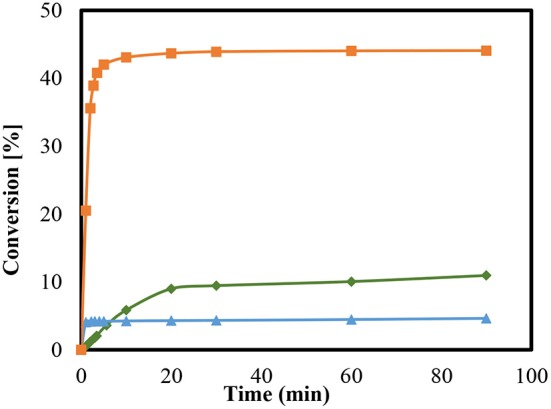
Conversions of condensation reaction between glycerol and acetone at room temperature using modified and unmodified MCM-41; MCM-41pure (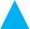), MCM 41- TD (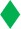), and MCM 41- SU (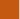).

Similarly, the zeolite-catalyzed condensation reactions with MCM-41 was used in various modifications for ketalization reaction. Every sample of the mesoporous material was examined at room temperature as is presented in Figure 6. The conversions are presented as a function of time for MCM41 (pure), MCM41-TD, and MCM 41-SU. As shown in Figure 6, the conversions are dependent on the degree of modification of the MCM-41. The silanol groups of the surface revealed higher catalytic activity than the untreated sample (pure MCM-41). These groups have a slightly acidic property and revealed up to 10% conversion. In contrast, the use of MCM41-SU in this reaction provided very low conversion and revealed up to around 5% after 90 min.

Conversely, the catalytic species of the mesoporous materials generated by introducing sulfonic acid groups with the aid of sulfuric acid yielded a remarkably higher condensation reaction. This particular conversion yielded almost 44%, after 90 min, which accords with the reaction equilibrium and subsequently indicating a high activity of the catalyst. The conversion of treated sample appears to be greater by comparison than that of pure MCM 41, which is much slower. The ketalization of glycerol over on sulfated sample (MCM 41- SU) may be catalyzed by these sulfate groups and any homogenous catalysis should add to the process of global catalysis. This appears to be the cause of the highest conversion of this catalyst in the case of sulphuric acid over MCM-41.

The literature (Li et al., 2012) also reported that both water and impurities combined to affect the activity of the sample. This may explain why higher catalytic activity was not observed with increased reaction time.

Researchers recently modified the sample of MCM-41 with vanadium for the Ketalization of glycerol (Abreu et al., 2019). The results (Abreu et al., 2019) showed that the conversion relies on the amount of acid sites engendered in the silica structure. Other literature (Yasmin and Müller, 2010) reported a varying range of modifications for MCM-41, with trifunctional alkylsilanes. These particular modifications (Yasmin and Müller, 2010) resulted in a better reduction of the physical properties of the sample (MCM-41) than those modified with monofunctional silanes (Yasmin and Müller, 2010).

Additionally, a high selectivity toward solketal was achieved using supported SiW and MCM-41 catalysts (30% SiW11/MCM-41, 30% SiW12/MCM-41) together with benzaldehyde (Narkhede and Patel, 2014). The results showed that the highest solketal selectivity of 82, with 85% glycerol conversion and at a 30°C room temperature could be achieved on the 30%-SiW11/MCM-41. It also showed that in 1 h, a 100 mg catalyst weight and a 1/1.2 molar ratio of glycerol to benzaldehyde are achievable. Additionally, an increase in the selectivity toward solketal was observed by adjusting the acidity levels of the parent SiW. Acidity strength together with larger pores and surface areas accounts for the high activity noted in these catalysts (Narkhede and Patel, 2014).

It is worth noting that in this current investigation, there was a decrease in the surface area values for all samples after the reaction that also included a loss in the microspore area. Higher catalytic activity was observed with a higher surface area on Zeolite ZSM-5 (160). However, the adsorption of the products on the catalysts surface led to a decrease in the BET surface areas in the range of 30–40 [m^2^/g]. It was also found that increasing the reaction time did not result in a proportionally higher glycerol conversion. This occurrence can be explained by the fact that in the acetalisation of acetone with glycerol, full conversion can be only obtained when either H_2_O is removed from the system or when acetone is used in large quantities.

### Kinetic Modeling of Zeolites Adsorption and Desorption Paths by the Condensation Reaction Between Glycerol and Acetone

**Figure 7:**
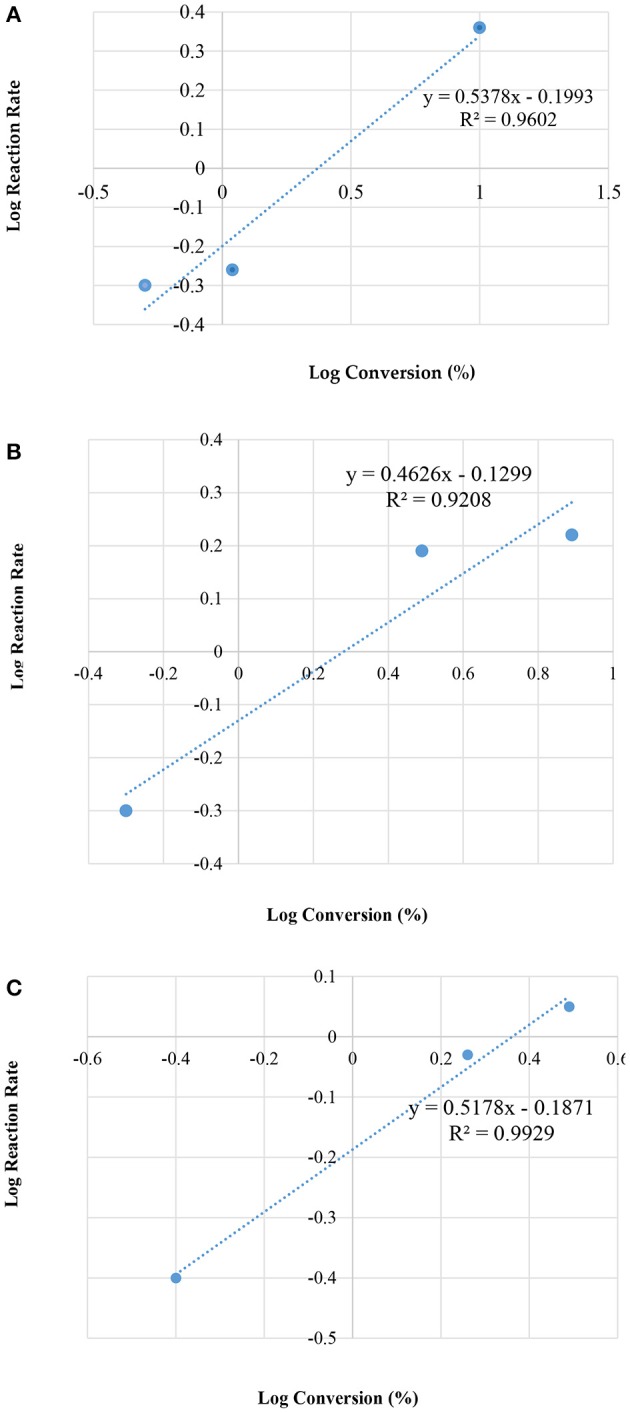
Plot of log reaction rate vs. log concentrations, for different modulus of HZSM-5; **(A)** 35; **(B)** 90; and **(C)** 160, by the condensation reaction between glycerol and acetone.

Figure 7 illustrates the kinetic energy for zeolite HZSM-5 with different modulus; 35, 90, and 160, respectively.

Table 4 presents the rate constant (k) and the overall rate (*n*), for the three different HZMS-5 zeolite and with different silica to alumina modules ratios at 25°C. The kinetic parameters were ascertained using only the method of initial rate. The standard procedure dictates that three points are sufficient to draw a straight line graphs. Over a period of 10 min the three points were obtained.

**Table 4:** Rate constant (k) and overall rate (*n*), for three types of HZMS-5 zeolite and with different silica to alumina modules (ratios) and at the reaction temperature of 25°C.

**HZSM-5 with different Si/Al modules**	**Kinetic rate constant (k) (Conc.^**1/2**^ min^**−1**^)**	**Reaction rate order (*n*)**
M = 35	0.6320	n = 1/2
M = 90	0.7415	n = 1/2
M = 160	0.6500	n = 1/2

In addition, the rate data showed that the overall rate equation follows the half order (*n* = 1/2). The following equation represents rate reaction for the initial rate from zero to 10 min together with and the rate equation:

$$\begin{array}{l} \frac{d[conversion]}{d[time]}=k_{1}{\left[Conversion\right]}^{n\;=\;1/2}\\ \frac{d[conversion]}{d[time]}=0.6745\;(min^{-1})\;{\left[Conversion\right]}^{1/2} \end{array}$$

After 10 min, the rate of desorption follows the average overall rate equation as in the following;

$$\begin{array}{l} \frac{\;d\left[conversion\right]}{d\left[Time\right]}=k_{2}{\left[Conversion\right]}^{n=-1}. \end{array}$$

### Effect of Temperature on the Kinetics Reaction Rate

For the reaction system with HZSM-5 samples were at different temperatures of 25, 50, and 60°C.

Table 5, shows kinetic constant (k) and, rate order (*n*) with HZSM-5 (M = 90), at different reaction temperatures.

**Table 5:** Kinetic constant (k) and rate order (n) over HZSM-5 (M = 90), at different reaction temperatures.

**Temperature (^**°**^C)**	**Kinetic rate constant (k) (Conc.^**1/2**^ min^**−1**^)**	**Reaction rate order (*n*)**
25	0.74478	*n* = 1/2
50	4.436 × 10^3^	*n* = −2.5
60	1.3256 × 10^2^	*n* = −1.3

It is interesting to note that the reaction at 25°C with a M = 90 sample gave the same rate law as presented in the current paper (Table 5).

The reaction systems at 50 and at 60°C yielded different orders (*n*) indicating that at these relatively high temperatures the reactions follow different reaction pathways for each respective temperature and in turn create more complexities in the reaction system mechanism. Reaction at 50°C, produced order n = −2.5 and the k = 4.436 × 10^3^ (min^−1^) whereas the reaction at 60°C yielded n = −1.3 and k = 1.3256^*^10^2^ (min^−1^).

The unanticipated values recorded are likely due to the behavior of acetone during the gaseous phase of the reaction. Acetone evaporates at 50 and at 60°C. As a result, the catalyst active sites become saturated and glycerol is impeded and adsorption onto the catalyst surface ceases.

The reason is that glycerol molecules are not taken up onto sites into which acetone molecules were previously adsorbed. Glycerol adsorption can only occur on vacant sites. Glycerol molecules become attached at a rate, which is inversely proportional to the concentration of the vacant sites existing on the catalyst surface. During the gaseous phase, there is one mole of glycerol and three of acetone present.

**Figure 8:**
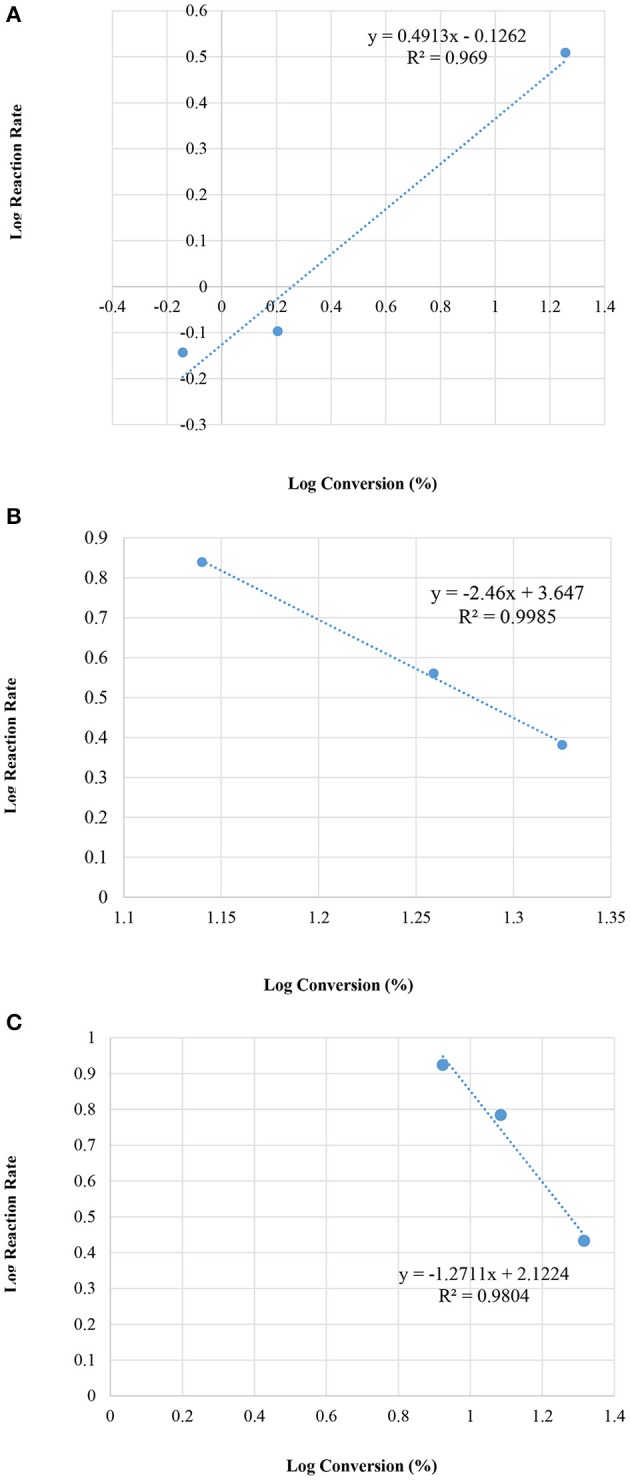
Plot of log initial reaction rate (before 10 min of the reaction) vs. log concentrations, at various reaction temperatures; **(A)** room temperature 25°C, **(B)** 50°C, and **(C)** 60°C.

Figure 8 illustrates the relation between the conversion and the initial reaction rate.

### Estimation of Arrhenius Energy (E_A_) for HZSM-5, at Various Reaction Temperatures (25, 50, and 60°C)

The Arrhenius energy E_A_ is calculated from the equations:

$$\begin{array}{l} \;k=k_{0}e^{-\frac{E_{A}}{RT}}\\ Ink=Ink_{0}-\frac{E_{A}}{R}\frac{1}{T} \end{array}$$

k = specific rate constant for a forward or reverse reaction (Conc.½ min^−1^). k_0_, pre-exponential factor; R, universal gas constant, in this case: 8.314 J mol^−1^ K^−1^; T, temperature in Kelvin (K); and E_A_, activation energy (kJ mol^−1^). A plot of $\frac{1}{T}$ vs. lnk gives the Arrhenius plot of the HZSM-5.

**Figure 9:**
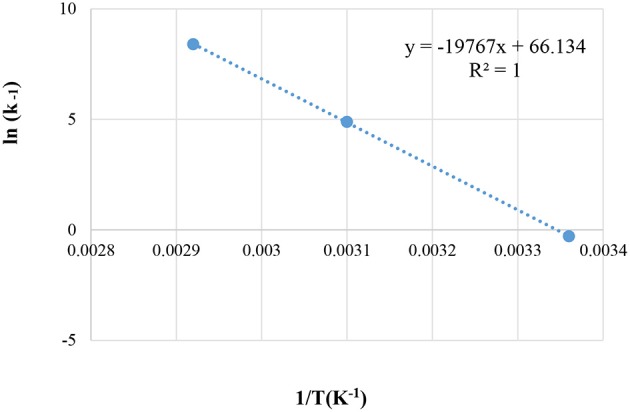
Arrhenius Energy (E_A_) for HZSM-5 at different reaction temperatures; of 25°C, 50°C, and 60°C.

The reaction temperatures are presented in Figure 9.

The activation energy using the liquid-phase ketalization of glycerol with acetone was also described in the literature (Esteban et al., 2015; Esposito et al., 2019). This current study found that an estimated E_A_, from the figure above is equal to 164.34 kJmol^−1^ and k_0_ = 5.2678^*^10^28^ (Con^1/2^. min^−1^). In the literature (Rossa et al., 2017) the activation energy (E_A_) for solkeltal reaction with H-BEA zeolite was calculated at reaction temperature ranging between 60 and 80°C and revealed Ea = 44.77 kJ mol^−1^ (Rossa et al., 2017). Conversely, in this current work, the reaction was performed at the lower reaction and temperatures ranging between 25 and 60°C. The result was a higher activation energy level of (164.34 kJ mol^−1^).

## Kinetic Study for Pure and Treated MCM-41 Samples

The reaction for the pure MCM- 41 seems to be very slow. The reaction itself is independent of the conversion, where n is zero (*n* = 0.1) and rate constant k = 0.5766 (Conc.½ min^−1^). Hence, more in-depth investigations are required to establish the reaction rate law. The reduced capacity to convert the acetone–glycerol adduct into solketal by Lewis acid sites over pure MCM 41 may in some measure explain the results obtained. This low rate is due to its hydrophobic property and to the absence of acidic centers (Rossa et al., 2017). The literature (Li et al., 2012) also reported that both water and impurities combined to affect the activity of the sample.

Moreover, the boiling point of Acetone is 56°C at one atmosphere, which corresponds to about 113.737 mm Hg vapor pressure. This saturates the active centers of the catalysts and reduces the efficiency of the catalysts within 10 min of reaction time.

**Figure 10:**
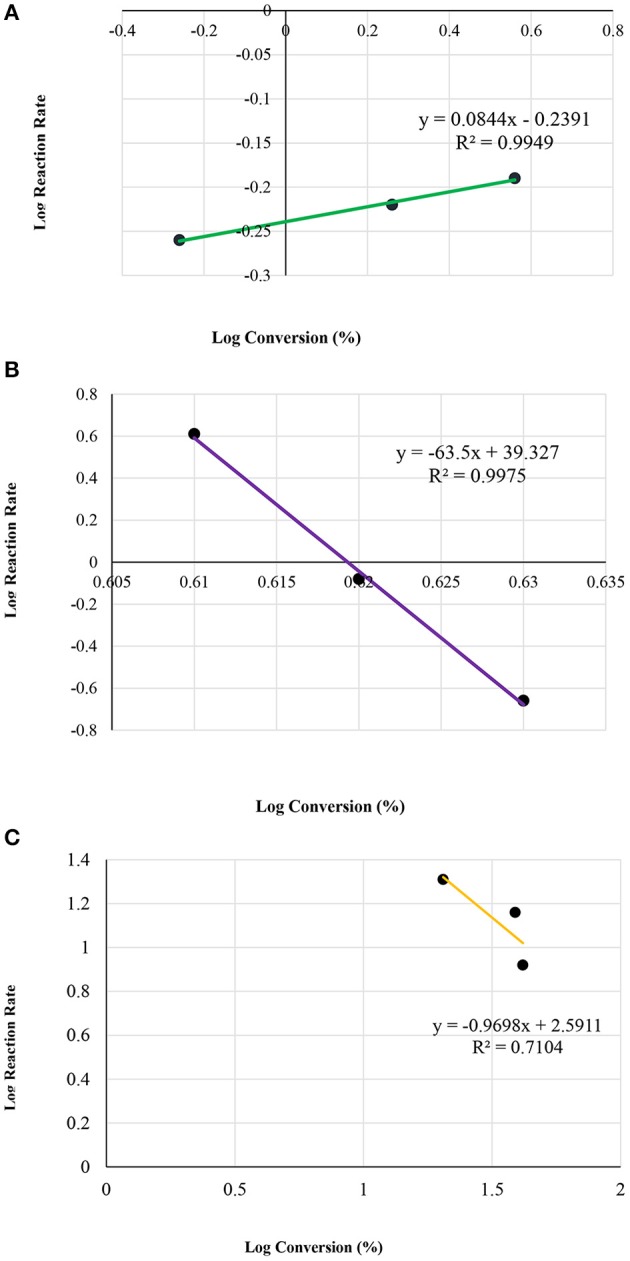
Plot of log initial reaction rate (before 10 min of reaction) vs. log concentrations, of the MCM-41 series; **(A)** MCM-41(pure); **(B)** MCM 41-TD, and **(C)** MCM 41-SU.

On the other hand, the MCM-41 Silanol reaction seemed to be saturated after 1 min following the reaction. Therefore, the rate law could not be determined. Furthermore, the sulfated MCM-41 presented the order of the reaction to be n = −1 and the rate constant, k = 3.9 ^*^ 10^2^ (Conc.½ min^−1^). Figure 10 presents an analysis of the kinetics data with the MCM-41 series from zero to 10 min.

## Kinetic Modeling of the Reaction System

### Reaction Rate Equation for the Ketalization Reaction, Without Effect of Water

The Solketal reaction presented in Figure 1 shows the overall reaction equation. With the kinetic study proposed, it is assumed that water has a significant effect on the reaction mechanism.

$$\begin{array}{l} Glycerol\;(reactant\;A)+Acetone\;\left(reactant\;B\right)\equiv \\ \;Solketal\;\left(product\;C\right)\\ \frac{d\left[C\right]}{dt}=k\left[A\right]\left[B\right]-k_{-1}\left[C\right] \end{array}$$

If: *K*_*eq*_ = *K*_equilibrium_ then:

$$\begin{array}{l} K_{eq}=\frac{\left[C\right]}{\left[A\right][B]}\\ \left[C\right]=\;(K_{eq})\left[A\right][B]\;=\;\frac{k_{1}}{k_{-1}}\left[A\right]\left[B\right] \end{array}$$

since;

$$\begin{array}{l} \frac{k_{1}}{k_{-1}}\;=K_{eq} \end{array}$$

then,

$$\begin{array}{l} \frac{d\left[C\right]}{dt}=k_{1}\left[A\right]\left[B\right]-K_{eq}\left[A\right]\left[B\right]\\ \frac{d\left[C\right]}{dt}=k_{1}\left[A\right]\left[B\right]-\frac{k_{1}}{k_{-1}}\;\left[A\right]\left[B\right]\\ \frac{d\left[C\right]}{dt}=k_{1}\left[A\right]\left[B\right]\;\left(1-\frac{1}{k_{-1}}\right) \end{array}$$

In general, if the molar ratio of acetone (reactant B) is higher than the molar ratio of Glycerol (reactant A):

$$\begin{array}{l} If\left[B\right]>\left[A\right]\\ k=k_{1}\left[B\right]\text{ and},\\ \frac{d\left[C\right]}{dt}=k\left[A\right]\left(1-\frac{1}{k_{-1}}\right) \end{array}$$

If: *k*_−1_ > *k* then;

$$\begin{array}{l} \left(1-\frac{1}{k_{-1}}\right)=1\\ \;\frac{d\left[C\right]}{dt}=k\left[A\right] \end{array}$$

The rate order for the current study as shown in Table 4 was found to be *n* = 1/2. The final equation then, can be generated as follows;

$$\begin{array}{l} \frac{d\left[C\right]}{dt}={k\left[A\right]}^{\frac{1}{2}} \end{array}$$

In general, the proposed kinetic model, as shown above, is in agreement with the literature (Rossa et al., 2017) where the only exception is that in the literature (Rossa et al., 2017), reaction temperature has occurred between 60 and 80°C and in the current investigation, reaction temperatures ranged between 25 and 60°C. In addition, order in the literature (Rossa et al., 2017) was found to be *n* = 1 and in the current study the rate order was estimated as *n* = 1/2 indicating that the reaction is very complex. This may be due to the fact to the catalyst surface is taking an active part in the reaction mechanism. Thereby, the relation between the surface area of the catalyst and absorption-desorption the mechanism should be more investigated in further detail.

### Reaction Rate Equation With Effect of Water

$$\begin{array}{l} Glycerol\;(reactant\;A)+Acetone\;\left(reactant\;B\right)\equiv \\ \;Solketal\;(product\;C)+Water\;\left(product\;D\right)\\ \frac{d\left[C\right]}{dt}=k\left[A\right]\left[B\right]-k_{-1}\left[C\right]\left[D\right] \end{array}$$

At equilibrium;

$$\begin{array}{l} \;\frac{d\left[C\right]}{dt}=0\\ k\left[A\right]\left[B\right]=k_{-1}\left[C\right]\left[D\right]\\ \;\frac{k_{1}}{k_{-1}}=\frac{\left[D\right]\left[D\right]}{\left[A\right]\left[B\right]}=k_{eq}\\ \;\left[C\right]\left[D\right]=k_{eq}\left[A\right]\left[B\right] \end{array}$$

Substitute in the first equation, we receive;

$$\begin{array}{l} \frac{d\left[C\right]}{dt}=k_{1}\left[A\right]\left[B\right]-k_{eq}\left[A\right]\left[B\right]\\ \left[A\right]\left[B\right]=k_{1}-k_{eq}\\ \frac{d\left[C\right]}{dt}=\left[A\right]\left[B\right]\left(k_{1}-\frac{k_{1}}{k_{-1}}\right)\\ \frac{d\left[C\right]}{dt}=k_{1}\left[A\right]\left[B\right]\left(1-\frac{1}{k_{1}}\right) \end{array}$$

In general, if the molar ratio of acetone (reactant B) is higher than the molar ratio of Glycerol (reactant A):

$$\begin{array}{l} \frac{d\left[C\right]}{dt}=k\left[A\right]\left(1-\frac{1}{k_{-1}}\right) \end{array}$$

The above final kinetics equation shows that water has no effect on the reaction from the point of view of kinetics. The current paper shows that the initial kinetics are calculated for a time reaction that occurs within the first 10 min. However, after these 10 min elapse, the reaction kinetics follow a different reaction equation with order *n* = −1. Water produced in the initial reaction during the first 10 min may well have a very significant role in the conversion activity.

In consequence, as the production of water is significant in the reaction system. The results obtained in this study are in agreement with the calculated kinetic parameters shown in Table 6 of the literature (Rossa et al., 2017), where for k_−1_ > k_1_, for the Ketalization reaction of Glycerol with Acetone using H-BEA catalyst at reaction temperatures between 40 and 80°C (Rossa et al., 2017).

**Table 6:** Kinetic parameter (k_1_ and k_−1_, L mol^−1^ min^−1^) responses calculated using R2W for the ketalization reaction of glycerol with acetone and the H-BEA catalyst (Rossa et al., 2017).

**Parameter**	**40^**°**^C**	**50^**°**^C**	**60^**°**^C**	**70^**°**^C**	**80^**°**^C**
k_1_	0.0082	0.0085	0.0082	0.0115	0.0213
k_−1_	0.0158	0.0159	0.0159	0.0205	0.0372
K_*eq*_	0.5159	0.5366	0.5179	0.5598	0.5720
X_A EXP_	76.01	75.17	74.16	74.52	75.54
X_A CAL_	70.70	71.16	70.80	71.90	72.00
X_*A eq CAL*_	70.75	71.18	70.81	71.90	72.05
Residue Q	254.22	154.77	193.22	100.50	56.58

*(X_A EXP_, X_CAL_, and X_A eq CAL_, %; Residue Q, mol L^−1^)*.

### Evaluation of Constant Equilibrium (**K**_**eq**_**)** With the Effect of Water Production

$$\begin{array}{l} Glycerol\;(reactant\;A)+Acetone\;\left(reactant\;B\right)\equiv \\ \;Solketal\;(product\;C)+Water\;\left(product\;D\right)\\ \frac{k_{1}}{k_{-1}}=\;k_{eq}=\;\frac{C_{c}\;*\;C_{D}}{C_{A}\;*\;C_{B}} \end{array}$$

For equal molar ratio of Glycerol and acetone: *C*_*A*0_ = *C*_*B*0_ the following initial equations apply:

$$\begin{array}{l} C_{A}=C_{A0}-C_{A0}\;X_{A0}\\ C_{B}=C_{B0}-C_{A0}\;X_{A0}\\ C_{C}=C_{C0}+C_{A0}\;X_{A0}\\ C_{D}=C_{D0}+C_{A0}\;X_{A0} \end{array}$$

where,

*C*_*A*0_, Initial Concentration of Glycerol (mol/L)
*C*_*B*0_, Initial Concentration of Acetone (mol/L)
*C*_*C*0_, Initial Concentration of solketal product (mol/L)
*C*_*D*0_, Initial Concentration of water produced (mol/L)
*C*_*A*_, Concentration of Glycerol (mol/L), at time t (min.)
*C*_*B*_, Concentration of Acetone (mol/L), at time t (min.)
*C*_*C*_, Concentration of solketal product (mol/L), at time t (min.)
*C*_*D*_, Concentration of water produced (mol/L), at time t (min.)
*X*_Aeq_, Equilibrium Conversion Glycerol.

Assuming that molar ratio of glycerol to molar ratio of acetone is 1:1, then:

$$K_{eq}\;=\;\frac{\left[C_{C0}+C_{A0}\;X_{Aeq}][C_{D0}+C_{A0}\;X_{A0}\right]}{\left[C_{A0}-C_{A0}\;X_{Aeq}][C_{A0}-C_{A0}\;X_{Aeq}\right]}$$

If *C*_*C*0_ = *C*_*D*0_ = 0

Then,

$$\begin{array}{l} K_{eq}\;=\;\frac{\left[C_{A0}\;X_{Aeq}][C_{A0}\;X_{A0}\right]}{\left[C_{A0}-C_{A0}\;X_{Aeq}][C_{A0}-C_{A0}\;X_{Aeq}\right]}\\ K_{eq}\;=\;\frac{{X_{Aeq}}^{2}}{{\left[1-X_{Aeq}\right]}^{2}}\\ K_{eq}{\left(1-X_{Aeq}\right)}^{2}=X_{Aeq}^{2}\\ K_{eq}\left(1-2X_{Aeq}+X_{Aeq}^{2}\right)\;=\;X_{Aeq}^{2}\\ K_{eq}-2K_{eq}X_{Aeq}+K_{eq}{X_{Aeq}}^{2}\;=\;X_{Aeq}^{2}\\ K_{eq}-2K_{eq}X_{Aeq}+(K_{eq})X_{Aeq}^{2}\;=\;X_{Aeq}^{2}\\ K_{eq}-2K_{eq}X_{Aeq}+(K_{eq}-1)X_{\text{Aeq}}^{2}\;=\;0\\ A=K_{eq}-1\\ B=-2K_{eq}\\ C=K_{eq}\\ X_{Aeq}\;=\;\frac{B\pm \sqrt{B^{2}-4AC}}{2A}\\ X_{Aeq}\;=\;\frac{2k_{eq}\;\pm \sqrt{{\left(-2K_{eq}\right)}^{2}-4\left(K_{eq}-1\right)\left(4K_{eq}\right)}}{2(K_{eq}-1)}\\ X_{Aeq}\;=\;\frac{2k_{eq}\;\pm \sqrt{4K_{eq}^{2}-4K_{eq}^{2}+4K_{eq}}}{2(K_{eq}-1)}\\ X_{Aeq}\;=\;\frac{2k_{eq}\;\pm 2\sqrt{K_{eq}}}{2(K_{eq}-1)} \end{array}$$

Then final proposed kinetic model generated is the following:

$$\begin{array}{l} X_{\text{Aeq}}=\;\frac{k_{eq}\;\pm \sqrt{K_{eq}}}{\left(K_{eq}-1\right)}. \end{array}$$

### Testing the Proposed Kinetic Model

The tabulated data in Table 6 obtained from the literature was used to test the proposed kinetic model (Rossa et al., 2017). The data is presented in Table 6 (Rossa et al., 2017) in its entirety for both reference and comparison (Rossa et al., 2017).

In order to achieve a valid comparison of the work done by Rossa et al. (2017), results in Table 6 above must be used at the current reaction conditions.

**Figure 11:**
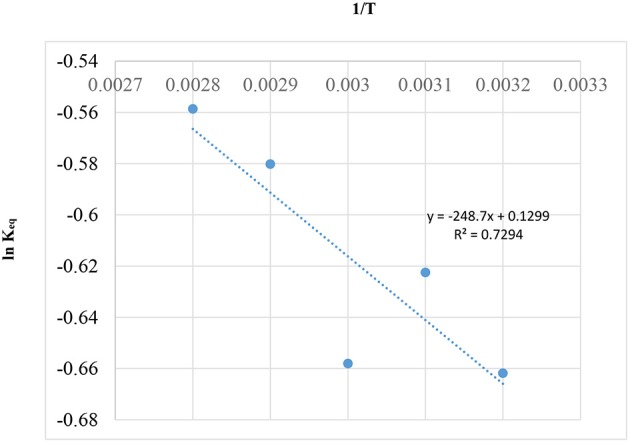
Plot of 1/*T* vs. ln K_eq_, using Gibbs free equation energy.

Since, catalyst will only have influence in the rate of reaction without affecting the equilibrium, a graph of 1/T vs. Ln K_eq_, using Gibbs free equation energy (Rossa et al., 2017), was drawn and is shown in Figure 11.

$$\begin{array}{l} \Delta \text{G}=-{\text{RTlnK}}_{\text{eq}} \end{array}$$

where,

ΔG, Gibbs free energy
T, temperature in Kelvin (K)
R, Gas constant, 8.314 JK^−1^ mol^−1^
K_*eq*_, Dissociation rate.

In Figure 11, the Regression (*R*^2^ = 0.7294) of the curve is not low, but it is very good for the scattered data in Table 6 of literature (Rossa et al., 2017). This positive result is possibly due to the reaction having occurred at a temperature above the boiling point of acetone (56°C). In this way, the behavior of the system is homogenous possibly due to the fact that the reaction is occurring during the gaseous phase and not on the catalytic surface.

In Figure 11, the equation representing the graph above (ln$\left(K_{eq}\right)=-248.2*\left(\frac{1}{T}\right)$ + 0.1299) can be used to generate *K*_*eq*_, *x*_*Aeq*_, and *t*_*eq*_ for reaction temperatures (25, 50, and 60°C) for the current study using the model equation in section Reaction Rate Equation for the Ketalization Reaction, Without Effect of Wateras in the following:

$$\begin{array}{l} \frac{d\left[C\right]}{dt}={k\left[A\right]}^{\frac{1}{2}} \end{array}$$

Where [A] = *C*_AO_-*C*_*AOeq*_ and $\frac{d\left[C\right]}{dt}\;=\;\frac{-C_{\text{AO}}dX_{\text{Aeq}}}{dt}$

$$\begin{array}{l} -C_{\text{AO}}\;\frac{dX_{\text{Aeq}}}{dt}\;=\;C_{\text{AO}}k\;\sqrt{\left(1-X_{\text{Aeq}}\right)}\\ \;-\;\frac{dX_{\text{Aeq}}}{\sqrt{\left(1-X_{eq}\right)}}=kt\\ \;t_{eq}=\frac{2\sqrt{1-X_{\text{Aeq}}}}{k\;} \end{array}$$

where, *t*_*eq*_ is the time for the reaction equilibrium to be obtained.

Applying the above kinetic proposed model for the current temperatures (25, 50, and 60°C); from Figure 9, *X*_Aeq_ and the equilibrium time (t_*eq*_) can be estimated and is shown in Table 7.

**Table 7:** Testing the proposed kinetic model and its correlation with the literature (Rossa et al., 2017).

**Temperature (^**°**^C)**	**Equilibrium constant (K_**eq**_)**	**Equilibrium conversion (XA_**eq**_)**	**Reaction equilibrium time teq (min)**
25	0.4830	0.4115	2.28
50	0.5348	0.4205	2.26
60	0.5488	0.4236	2.25

It has been found that the estimated reaction equilibrium time (t_*eq*_) is within the range from zero to 10 min and, is in agreement with the proposed kinetic model in section Kinetic Modeling of the Reaction System. It was determined that the estimated reaction equilibrium time (*t*_*eq*_) is within the range of zero to 10 min and this agrees with the proposed kinetic model in Section Reaction Rate Equation for the Ketalization Reaction, Without Effect of Wateras (*k*_−1_ > *k*) of the current study.

Table 8 shows a comparison of the k_1_, equilibrium constant and equilibrium conversion values contained in the current study with those by Rossa et al. as shown in Table 6 (Rossa et al., 2017).

**Table 8:** Comparison of the k_1_ values, equilibrium constant values, and equilibrium conversion values of the present study and values from Rossa et al. (2017).

**Temperature (^**°**^C)**	**Rate constant (k**_****1****_**)**		**Equilibrium constant (K**_********eq********_**)**		**Equilibrium conversion (X**_*******A eq*******_**)**	
	**Present work (Conc.^**1/2**^ min^**−1**^)**	**Rossa et al. (2017) (Conc.^**−1**^ min^**−1**^)**	**Present work (Calculated)**	**Rossa et al. (2017)**	**Present work (Calculated)**	**Rossa et al. (2017)**
25	0.74478	–	0.4830	–	0.4115	–
50	4.436 × 10^3^	0.0085	0.5348	0.5366	0.4205	0.7517
60	1.3256 × 10^2^	0.0082	0.5488	0.5279	0.4236	0.7452

Table 8 above shows that the equilibrium constant **(K**_***eq***_**)** at temperatures 50 and 60°C are within the same order of magnitude. In this work, a low equilibrium conversion **(X**_**Aeq**_**)** approximately 0.42 (42%) was obtained.

Meanwhile, Rossa et al. (2017) report a relatively high equilibrium conversion reaching up to 0.75 (75%). The sizeable difference between the two results can be accounted for when considering a number of factors; the reaction environment, the catalyst type including its physical and chemical properties and the turnover frequency (TOF). This is a quantitative measure of the activity of the catalyst.

However, for this current study, the turnover frequency (TOF) was not measured since a large quantity of the reactant acetone employed for solketal production would be in a gaseous state at temperatures of 50 and 60°C.

By definition the Turnover Frequency (TOF) is used to quantify the activity of the catalyst. It refers to the number of reacting molecules per active site per second at the condition of the experiment (Scott Fogler, 2006). When a metal catalyst is deposited on a support, the metal atoms are considered active sites (Nda-Umar et al., 2018).

Thus,

$$\begin{array}{l} r_{M}^{,}=f_{Product}\;*\;D\;*\left(\frac{1}{MW_{Metal}}\right)\left(\frac{\%\;Metal}{100}\right) \end{array}$$

where:

$r_{M}^{,},\;$the rate of formation of product turnover frequency.

*f*_*Product*_: Turnover Frequency 3.1 min^−1^ = 0.052 s^−1^ at one bar (1 atmosphere) (Nda-Umar et al., 2018).

Usually, the Turnover Frequency is calculated based on the glycerol conversion/^*^ product yield per gram catalyst per total reaction time.

D, The dispersion of the catalyst as a fraction of metal atom deposited on the surface.

*MW*_Metal_, The Molecular Weight of Metal deposited on the surface = $Al_{\text{MWt}}\;=27\frac{g}{mol}$

*%Metal*, The catalysts dispersion percentage of atoms exposed and determined from the reactant chemisorption.

In the current study, the number in the parenthesis in [(SiO_2_)x(Al_2_O_3_)y] corresponds to the ratio of the oxides, the so called module; SiO_2_/Al_2_O_3_ (usually this value is twice as high as the Si/Al ratio). Hence, it is important to mention that the value “percentage dispersion of alumina on silicon” is quite unusual because of Aluminum (Al), as single atom and due to the Loewenstein's rule, there are no Al-O-Al bonds. The Aluminum (Al) is randomly distributed in silica. For example, each atom of Aluminum creates a negative charge which is compensated by counter-ion, e.g., Na^+^ or H^+^. The number of Aluminum (Al) determines the number of acidic sites. Hence, when the weight of the catalyst is ascertained, the amount of Aluminum can be calculated.

The molecular weight Al_2_O_3_ is 102 [g/mol], and the molecular weight SiO_2_ is 60.081 [g/mol]. From that, the dispersion or the distribution of Al on the catalyst surface of a sample like for example, ZSM-5 (35) can be calculated as the following:

*n* = **35** * 60.081 g (SiO_2_) + 1 * 102 g (Al_2_O_3_) = 2204.835 g, i.e., 712 g zeolite contains 54 g Aluminum (because of the 2 atoms in the oxide). Therefore, 1 g (water free) zeolite contains:

(54*1/2204.835) = 0.0245 g Aluminum and, 1 g (water free) zeolite contains (28*1/2204.835) = 0.013 g Silicon. As a result, the dispersion or the distribution of Al on the catalyst surface $=\;\left(\frac{0.0245}{0.0245+0.013}\right)*100\;=\;65.3\%\;Al-metal$.

On the other hand, three mechanisms predominate in the catalytic reaction process; Adsorption, Catalytic Surface reaction and Desorption processes. For the present study, the Langmuir-Hinshelwood approach for determining the catalytic and heterogeneous mechanism is employed in order to illustrate the catalytic reaction process involved in solketal production from glycerol and acetone that is to say;

$$\begin{array}{l} Glycerol[A]+Acetone\left[B\right]=Solketal[C]+Water\left[D\right] \end{array}$$

The following adsorption mechanism is envisaged;

$$\begin{array}{l} \;\left\{\begin{array}{c} A+S↔A.S\\ B+S↔B.S \end{array}\right\}\text{ Fast}\\ \left\{A.S+B.S\;↔C.S+D.S\right\}\text{ Slow and rate limiting step}\\ \;\left\{\begin{array}{c} C.S↔C+S\\ D.S↔D+S \end{array}\right\}\text{ Fast} \end{array}$$

where *S* represents the surface of catalyst.

From any Chemical Engineering standard textbook (Scott Fogler, 2006) and using the method of initial rate and when products C and D are present:

$$\begin{array}{l} {-r}_{A}^{,}=\;k_{A}P_{A}P_{B} \end{array}$$

where;

*P*_*A*_, Partial pressure of Glycerol

*P*_*B*_, Partial pressure of Acetone

Under the experimental conditions of the present study *P*_*B*_ ≫ *P*_*A*_

So *k*_*A*_*P*_*B*_ = *k* and the initial rate of reaction becomes:

$$\begin{array}{l} {-r}_{A}^{,}\;=\;kP_{A}\\ \;P_{A}\;=\;P_{Total}\;*\;x_{A} \end{array}$$

where;

*P*_Total_ = System total Pressure

*x*_*A*_ = mole fraction of glycerol in the reaction system.

The initial rate equation in terms of fractional conversion is given by

$$\begin{array}{l} {-r}_{A}^{,}={kP}_{\text{Total}}\;*\;x_{A} \end{array}$$

and; *k*_Avg_ = *kP*_Total_ gives:

$$-r_{A}^{,}=k_{Avg}\;*\;x_{A}.$$

It is surmised that the mechanism governing steps like Adsorption process, Catalytic Surface reaction process and Desorption process are fast functioning relative to those remaining others outlined in the list. Mass transfer activity does not affect the overall reaction rate since the concentration of the surrounding area of the active sites are indistinguishable from those of the bulk fluid. In one study (Stawicka et al., 2016), both glycerol and the ratio of acetone molar substantially affected the kinetics and thermodynamics of the reaction in a procedure to condense glycerol with acetone (Stawicka et al., 2016). Molar of acetone to glycerol ratios reached 1,48:1, and 2.46:1 and in turn produced solketal yields of 68 and 74, respectively (Nanda et al., 2014a).

Current literature (Nanda et al., 2014b; Esteban et al., 2015) shows models for the pseudo-homogeneous (Esteban et al., 2015) and heterogeneous models (Nanda et al., 2014b; Esteban et al., 2015). However, no homogeneous model exists showing the ketalization reaction of glycerol with acetone. In spite of this, a pseudo-homogeneous model was proposed yielding values of k_−1_ > k_1_ using sulfonated resin as a catalyst. Water that is formed in the product must be extracted before equilibrium can be established. For this reason, there should be no additional water in the reaction system before a catalyst is employed. The pseudo-homogeneous model works best if any analysis is conducted at temperatures exceeding the boiling point (Esteban et al., 2015). Since 56°C is the boiling point of Acetone, this model will yield accurate results from reactions conducted in temperatures in temperatures that are in excess of it. This is apparent even when taking into consideration the reaction temperatures when they are close to, even exceeding the boiling point of acetone.

Excessive glycerol conversions, when treated with acetone, were the subject of another study by Ferreira et al. (2010). An increase in the glycerol to acetone molar ratio from 1:3 to 1: 12 coincided with an improvement in the glycerol conversion. However, the selectivity of solketal stayed the same (Ferreira et al., 2010).

Moreover, a reaction equation involving water equilibrium (*K*_*w*_) is proposed in the literature (Li et al., 2012; Nanda et al., 2016). However, for this study a water equilibrium is not especially important. For reasons discussed previously in section Kinetic Modeling of the Reaction System, as significant quantities of water are produced, adverse reactions immediately occur in the main product. In addition, reverse, as opposed to forward reaction is faster and therefore water equilibrium does not have any special role. By this reasoning and those suggested in references (Ferreira et al., 2010; Reddy et al., 2011; Royon et al., 2011; Ortiz et al., 2012; Menezes et al., 2013; Nanda et al., 2014b; Aghbashlo et al., 2018, 2019; Ammaji et al., 2018; Fatimah et al., 2019), water equilibrium is not reached nor is it considered necessary for this current proposed kinetic model. Since water is formed during the experimental reaction process, experimentally the effect of water has not been studied in the current work.

Moreover, at a reaction temperature of 60°C (333.15 K) and at one atmosphere, the acetone was shown to exert a vapor pressure of about 113.737 mm Hg. Hence, the overall order of the reaction was determined by the method of initial rates.

## Conclusions

In summary, the catalytic effect of HZSM-5 with a variety of silica to alumina ratios were investigated. The conversions ranged between 30% to around 38%. More significantly, the current research was able to establish the rate law for HZMS-5 zeolite with different silica to alumina ratios (M = 35, 90, and 160), the ‘n’ order equal to half and with an average rate constant *k* = 0.6745 (Conc.½ min^−1^). Additionally, the results of the Arrhenius plots for HZSM-5 at different reaction temperatures (25, 50, and 60°C) showed activation energy of E_A_ = 164.34 164.34 kJmol^−1^ and k_0_ = 5.2678^*^10^28^ (min^−1^). Furthermore, the results conclude that the reaction with pure MCM- 41 occurs very slowly, and that the reaction also occurs independent of the conversion, where ‘n’ is zero (*n* = 0.1) and rate constant *k* = 0.5766 (Conc.½ min^−1^).

Henceforth, more in depth investigation is needed in order to establish the reaction rate law. The rate of the reaction equation for the system ketalization reaction was established with the effect of production of water but again without a significant water effect on the reaction system. To end, the equilibrium time for the reaction system was observed as constant with an average duration of around 3 min (≈ 2.26 min).

Furthermore, a mathematical approach has been proposed in this current work to calculate the dispersion or the distribution of Al on the catalyst surface like for example ZSM-5 that has different modules [i.e., different molar ratios of (SiO_2_/Al_2_O_3_)].

## Data Availability Statement

All datasets generated for this study are included in the article/supplementary material.

## Author Contributions

MA analyzed and interpreted the data and wrote the paper.

### Conflict of Interest

The author declares that the research was conducted in the absence of any commercial or financial relationships that could be construed as a potential conflict of interest.
